# Differential impact of fentanyl and morphine doses on ticagrelor-induced platelet inhibition in ST-segment elevation myocardial infarction: a subgroup analysis from the PERSEUS randomized trial

**DOI:** 10.3389/fcvm.2024.1324641

**Published:** 2024-04-02

**Authors:** Dorian Garin, Sophie Degrauwe, Federico Carbone, Yazan Musayeb, Nathalie Lauriers, Marco Valgimigli, Juan F. Iglesias

**Affiliations:** ^1^ Department of Cardiology, Geneva University Hospitals, Geneva, Switzerland; ^2^ Department of Internal Medicine, First Clinic of Internal Medicine, University of Genoa, Genoa, Italy; ^3^ IRCCS Ospedale Policlinico San Martino Genoa, Italian Cardiovascular Network, Genoa, Italy; ^4^ Department of Cardiology, Lausanne University Hospital, Lausanne, Switzerland; ^5^ Istituto Cardiocentro Ticino, Ente Ospedaliero Cantonale, Lugano, Switzerland

**Keywords:** fentanyl, dose, pharmacodynamics, pharmacokinetics, ST-segment elevation myocardial infarction, ticagrelor

## Abstract

**Introduction:**

Among patients with ST-segment elevation myocardial infarction (STEMI) treated with primary percutaneous coronary intervention (PCI), intravenous fentanyl does not enhance ticagrelor-induced platelet inhibition within 2 h compared to morphine. The impact of the total dose of fentanyl and morphine received on ticagrelor pharmacodynamic and pharmacokinetic responses in patients with STEMI remains however undetermined.

**Materials and methods:**

We performed a post-hoc subanalysis of the prospective, open-label, single-center, randomized PERSEUS trial (NCT02531165) that compared treatment with intravenous fentanyl vs. morphine among symptomatic patients with STEMI treated with primary PCI after ticagrelor pretreatment. Patients from the same population as PERSEUS were further stratified according to the total dose of intravenous opioids received. The primary outcome was platelet reactivity using P2Y_12_ reaction units (PRU) at 2 h following administration of a loading dose (LD) of ticagrelor. Secondary outcomes were platelet reactivity and peak plasma levels of ticagrelor and AR-C124910XX, its active metabolite, at up to 12 h after ticagrelor LD administration. Generalized linear models for repeated measures were built to determine the relationship between raw and weight-weighted doses of fentanyl and morphine.

**Results:**

38 patients with STEMI were included between December 18, 2015, and June 22, 2017. Baseline clinical and procedural characteristics were similar between low- and high-dose opioid subgroups. At 2 h, there was a significant correlation between PRU and both raw [regression coefficient (B), 0.51; 95% confidence interval (CI), 0.02–0.99; *p* = 0.043] and weight-weighted (B, 0.54; 95% CI, 0.49–0.59; *p* < 0.001) doses of fentanyl, but not morphine. Median PRU at 2 h was significantly lower in patients receiving low, as compared to high, doses of fentanyl [147; interquartile range (IQR), 63–202; vs. 255; IQR, 183–274; *p* = 0.028], whereas no significant difference was found in those receiving morphine (217; IQR, 165–266; vs. 237; IQR, 165–269; *p* = 0.09). At 2 h, weight-weighted doses of fentanyl and morphine were significantly correlated to plasma levels of ticagrelor and AR-C124910XX.

**Conclusion:**

In symptomatic patients with STEMI who underwent primary PCI after ticagrelor pretreatment and who received intravenous opioids, we found a dose-dependent relationship between the administration of intravenous fentanyl, but not morphine, and ticagrelor-induced platelet inhibition.

## 1 Introduction

Early initiation of dual antiplatelet therapy (DAPT) with aspirin and a potent orally administered P2Y_12_ receptor inhibitor is the mainstay of pharmacological management for patients with ST-segment elevation myocardial infarction (STEMI) treated with primary percutaneous intervention (PCI) to reduce adverse ischemic and thrombotic events (1). However, in the setting of acute STEMI, platelet inhibition elicited by potent oral P2Y_12_ receptor antagonists is delayed due to the adverse hemodynamic conditions and delayed gastro-intestinal absorption (2, 3). High on-treatment platelet reactivity (HTPR) after oral P2Y_12_ receptor inhibitor administration has been shown to increase the risk of major adverse ischemic outcomes, including death, myocardial infarction, and stent thrombosis among patients with acute coronary syndrome (ACS) who underwent PCI (4).

Intravenous morphine, which is widely used during STEMI management to relieve acute pain and anxiety, further impairs the antiplatelet response to potent orally administered P2Y_12_ receptor inhibitors due to delayed gastro-intestinal drug absorption (5, 6), which may be further potentiated by nausea and vomiting resulting from morphine administration (7, 8). During the last years, several strategies aimed at achieving earlier platelet inhibition among STEMI patients undergoing primary PCI have been investigated, such as alternative routes (9–13) and timing (14) of P2Y_12_ receptor inhibitors administration, the use of different opioid (15) and non-opioid (16) analgesic agents, and the concomitant use of prokinetic agents (17) or peripheral opioid antagonists (18) to promote gastro-intestinal motility. In the PERSEUS (“*Platelet Inhibition after Pre-hospital Ticagrelor using Fentanyl compared to Morphine in patients with ST-segment elevation Myocardial Infarction undergoing Primary Percutaneous Coronary Intervention”)* randomized trial, intravenous fentanyl administration failed to improve platelet inhibition induced by ticagrelor within 2 h as compared to morphine among symptomatic STEMI patients treated with primary PCI, despite a signal suggesting improved ticagrelor bioavailability and more potent platelet inhibition with fentanyl compared to morphine (15, 19). Recent studies have suggested a dose-dependent relationship between intravenous opioids administered and platelet inhibition induced by potent oral P2Y_12_ receptor antagonists in patients treated with PCI (20, 21). Higher doses of morphine have been shown to significantly reduce ticagrelor absorption and attenuate its antiplatelet effects as compared to lower morphine doses among patients with STEMI treated with primary PCI (20). However, potential differences in the effects of total doses of fentanyl vs. morphine received on platelet inhibition elicited by orally administered P2Y_12_ inhibitors among patients with STEMI have never been reported to date. Therefore, we sought to compare the impact of fentanyl and morphine doses received on the pharmacodynamic and pharmacokinetic profiles of ticagrelor among patients with STEMI treated with primary PCI.

## 2 Materials and methods

The present study is a *post-hoc* subgroup analysis of the PERSEUS prospective, single-centre, open-label, randomized controlled trial. Details on the rationale and design of the PERSEUS trial have been previously reported (22). In summary, the same population as PERSEUS of patients with acute STEMI planned to undergo primary PCI and who required intravenous opioids for pain relief [Visual Analog Scale Score (VAS) ≥ 3] were randomized (1:1) to receive fentanyl (50–150 *µ*g) or morphine (4–8 mg) after pretreatment with intravenous aspirin (500 mg) and ticagrelor (180 mg). Subsequent doses of intravenous opioids were administered to achieve a VAS <3. Exclusion criteria included prior use of P2Y_12_ receptor inhibitors or anticoagulants before STEMI diagnosis, administration of glycoprotein IIb/IIIa antagonists, and the presence of conditions affecting gastro-intestinal absorption or metabolism of oral P2Y_12_ receptor inhibitors, such as cardiogenic shock. Comatose patients were also excluded. The PERSEUS study protocol complied with the Declaration of Helsinki and received approval from the Ethics Committee at Lausanne University Hospital, Switzerland. Written informed consent was obtained from all participants. The trial was registered with ClinicalTrials.gov, identifier *NCT02531165*. The results of the overall patient population included in the PERSEUS trial have been reported elsewhere (15, 19).

### 2.1 Pharmacodynamic and pharmacokinetic assessments

Complete details on ticagrelor pharmacodynamic and pharmacokinetic data collection methods were described previously (22). We assessed platelet reactivity by P2Y_12_ reaction units (PRU) using the VerifyNow® P2Y_12_ function test (*Accumetrics, Inc., San Diego, California, USA*). A blinded external laboratory (*Covance Laboratories, Indianapolis, Indiana, USA*) measured plasma levels of ticagrelor and its active metabolite (AR-C124910XX) at 1, 2, 4, 6, and 12 h after ticagrelor LD administration. Plasma concentrations of ticagrelor and AR-C124910XX before ticagrelor LD were assumed to be 0 mg/ml because patients on chronic P2Y_12_ inhibitor treatment were excluded.

### 2.2 Study outcomes

The primary outcome of this study was platelet reactivity measured by PRU, according to the total dose of intravenous opioids (fentanyl vs. morphine) received at 2 h following ticagrelor pretreatment. The secondary outcomes, based on the total dose of intravenous opioids given, were (1) PRU at 1, 4, 6, and 12 h and (2) maximum plasma concentrations of ticagrelor and AR-C124910XX at 1, 2, 4, 6, and 12 h post-ticagrelor LD administration.

### 2.3 Statistical analysis

This is a post-hoc, non-prespecified, subgroup analysis from the PERSEUS randomized controlled trial. Patients from the total study cohort who were randomly allocated to receive intravenous fentanyl or morphine were further divided into two subgroups (low vs. high) according to the total dose of intravenous opioids received. Low vs. high doses subgroups were defined according to the total dose of intravenous opioids received below vs. above the median value, respectively. Mann-Whitney and Fisher exact tests were used, when appropriate. Generalized linear models (GLM) using B (regression coefficient) and corresponding 95% confidence intervals (CI) were built to estimate interactions between the randomized opioid treatment and time at different timepoints following ticagrelor LD administration. B coefficients represented the change in the dependent variable for a one-unit change in the independent variable, while holding other variables constant. Both raw and weight-weighted doses of intravenous opioids were integrated as independent variables to determine their impact on dependent variables. The raw dose corresponded for the absolute quantity of intravenous opioid received, whereas the “weight-weighted” dose adjusted the raw dose received per kilogram of patient body weight. A *p* value < 0.05 was considered as statistically significant. All statistical analyses were performed using IBM SPSS Statistics, Version 23.0 (*IBM CO., Armonk, NY*) and GraphPad Prism 5 (*GraphPad Software, Inc, La Jolla, CA*).

## 3 Results

### 3.1 Baseline characteristics

Between December 18, 2015, and June 22, 2017, 38 patients with STEMI were included in the PERSEUS trial, of which 19 patients were treated with fentanyl and 19 patients received morphine. For this new analysis, no patient was excluded. Patient baseline clinical and procedural characteristics in the subgroups receiving low vs. high doses of fentanyl and morphine are reported in Table 1. There were no significant differences between the two treatment arms with the exception of higher rates of left anterior descending artery and lower rates of right coronary artery involvements in the low vs. high fentanyl dose subgroup.

**Table 1:** Patient baseline clinical and angiographic characteristics in low and high fentanyl vs. morphine doses groups.

	Fentanyl			Morphine		
Characteristics	Low dose (*n* = 10)	High dose (*n* = 8)	*p*-value	Low dose (*n* = 10)	High dose (*n* = 9)	*p*-value
Age, years [IQR]	70 (59–82)	68 (52–82)	0.762	72 (60–81)	54 (46–74)	0.156
Male sex, *n* (%)	8 (80.0)	4 (50.0)	0.321	8 (80.08)	6 (66.7)	0.628
Weight, kg [IQR]	84 (63–92)	75 (63–79)	0.173	75 (73–89)	80 (76–95)	0.278
BMI, kg/m^2^ [IQR]	27.7 (21.1–30.3)	25.3 (22.7–27.9)	0.573	25.7 (23.0–27.8)	26.3 (23.9–30.5)	0.315
Hypertension, *n* (%)	5 (50.0)	3 (37.5)	0.516	5 (50.0)	5 (55.6)	0.999
Dyslipidemia, *n* (%)	3 (42.9)	4 (50.0)	0.630	5 (50.0)	3 (33.3)	0.650
Diabetes mellitus, *n* (%)	2 (20.0)	1 (12.5)	0.999	1 (10.0)	2 (22.2)	0.582
Smoking						
Active, *n* (%)	3 (30.0)	3 (37.5)	0.999	3 (30.0)	3 (33.3)	0.999
Former, *n* (%)	5 (50.0)	1 (12.5)	0.152	2 (20.0)	0 (0.0)	0.474
Never, *n* (%)	2 (20.0)	4 (50.0)	0.321	5 (50.0)	5 (55.6)	0.999
Prior coronary artery disease	0 (0.0)	1 (12.5)	0.444	3 (30.0)	0 (0.0)	0.211
Prior myocardial infarction	1 (10.0)	1 (12.5)	0.358	4 (40.0)	1 (11.1)	0.073
Prior PCI	0 (0.0)	0 (0.0)	–	3 (30.0)	0 (0.0)	0.211
Chronic kidney disease	0 (0.0)	1 (12.5)	0.444	0 (0.0)	0 (0.0)	–
Cardiogenic shock	1 (10.0)	1 (12.5)	0.999	1 (10.0)	0 (0.0)	0.999
Infarct-related coronary vessel			0.018			0.251
Left main artery	0 (0.0)	0 (0.0)		0 (0.0)	1 (11.1)	
Left anterior descending artery	6 (60.0)	1 (12.5)		4 (40.0)	4 (44.4)	
Left circumflex artery	3 (30.0)	1 (12.5)		3 (30.0)	4 (44.4)	
Right coronary artery	1 (10.0)	6 (75.0)		3 (30.0)	0 (0.0)	
TIMI flow grade pre-PCI			0.407			0.201
0	6 (60.0)	5 (62.5)		9 (90.0)	7 (77.8)	
1	0 (0.0)	1 (12.5)		0 (0.0)	0 (0.0)	
2	2 (20.0)	2 (25.0)		1 (10.0)	0 (0.0)	
3	2 (20.0)	0 (0.0)		0 (0.0)	2 (22.2)	

Values are *n* (%), or median [interquartile range, IQR]. BMI, body mass index; PCI, percutaneous coronary intervention; TIMI, thrombolysis in myocardial infarction.

### 3.2 Pharmacodynamic assessment

Pharmacodynamic and pharmacokinetic profiles of ticagrelor and its active metabolite AR-C124910XX according to raw- vs. weight-weighed doses of fentanyl and morphine are summarized in Table 2. Using GLM, there was a significant relationship between PRU at 2 h and both raw (B, 0.51; 95% CI, 0.02–0.99; *p* = 0.043) and weight-weighted (B, 0.54; 95% CI, 0.49–0.59; *p* < 0.001) doses of fentanyl, but not morphine (B, 1.73; 95% CI, −8.33–11.79; *p* = 0.728, and B, 0.91; 95% CI, −0.08–1.92; *p* = 0.066, respectively) (Table 2). Similarly, both raw (B, 0.49; 95% CI, 0.04–0.93; *p* = 0.034) and weight-weighted (B, 0.52; 95% CI, 0.47–0.57; *p* < 0.001) doses of fentanyl were significantly correlated with PRU at 1 h following ticagrelor LD administration. There was no significant relationship between PRU and raw doses of morphine (B, −0.35; 95% CI, −9.62–8.92; *p* = 0.939), whereas a negative relationship was observed between PRU and weight-weighted doses of morphine (B, −1.10; 95% CI, −2.02–−0.17; *p* = 0.016) (Table 2).

**Table 2:** Pharmacodynamic and pharmacokinetic profiles of ticagrelor and its active metabolite AR-C124910XX according to raw- vs. weight-weighed doses of fentanyl and morphine.

	Fentanyl				Morphine			
	Raw-dose		Weight-weighted dose		Raw-dose		Weight-weighted dose	
	B (95% CI)	*p*-value	B (95% CI)	*p*-value	B (95% CI)	*p*-value	B (95% CI)	*p*-value
PRU								
At 1 h	0.49 (0.04–0.93)	0.034	0.52 (0.47–0.57)	<0.001	−0.35 (−9.62–8.92)	0.939	−1.10 (−2.02 – −0.17)	0.016
At 2 h	0.51 (0.02–0.99)	0.043	0.54 (0.49–0.59)	<0.001	1.73 (−8.33–11.79)	0.728	0.91 (−0.08–1.92)	0.066
Ticagrelor concentration								
At 1 h	−1.18 (−2.69–0.34)	0.123	−1.18 (−1.34 – −1.01)	<0.001	1.23 (−29.10–31.56)	0.935	2.07 (−0.99–5.13)	0.185
At 2 h	−1.40 (−3.30–0.49)	0.142	−1.39 (−1.60 – −1.18)	<0.001	−13.85 (−51.82–24.12)	0.463	−13.47 (−17.26 – −9.68)	<0.001
AR-C124910XX concentration								
At 1 h	−0.14 (−0.33–0.05)	0.145	−0.14 (−0.16 – −0.12)	<0.001	0.52 (−3.27–4.31)	0.782	0.66 (0.28–1.04)	0.001
At 2 h	−0.26 (−0.59–0.07)	0.113	−0.27 (−0.31 – −0.24)	<0.001	−1.25 (−7.86–5.35)	0.702	−1.10 (−1.77 – −0.43)	0.001

B, regression coefficient for the predictor variable from generalized linear models; CI, confidence interval; PRU, platelet reactivity unit.

**Figure 1:**
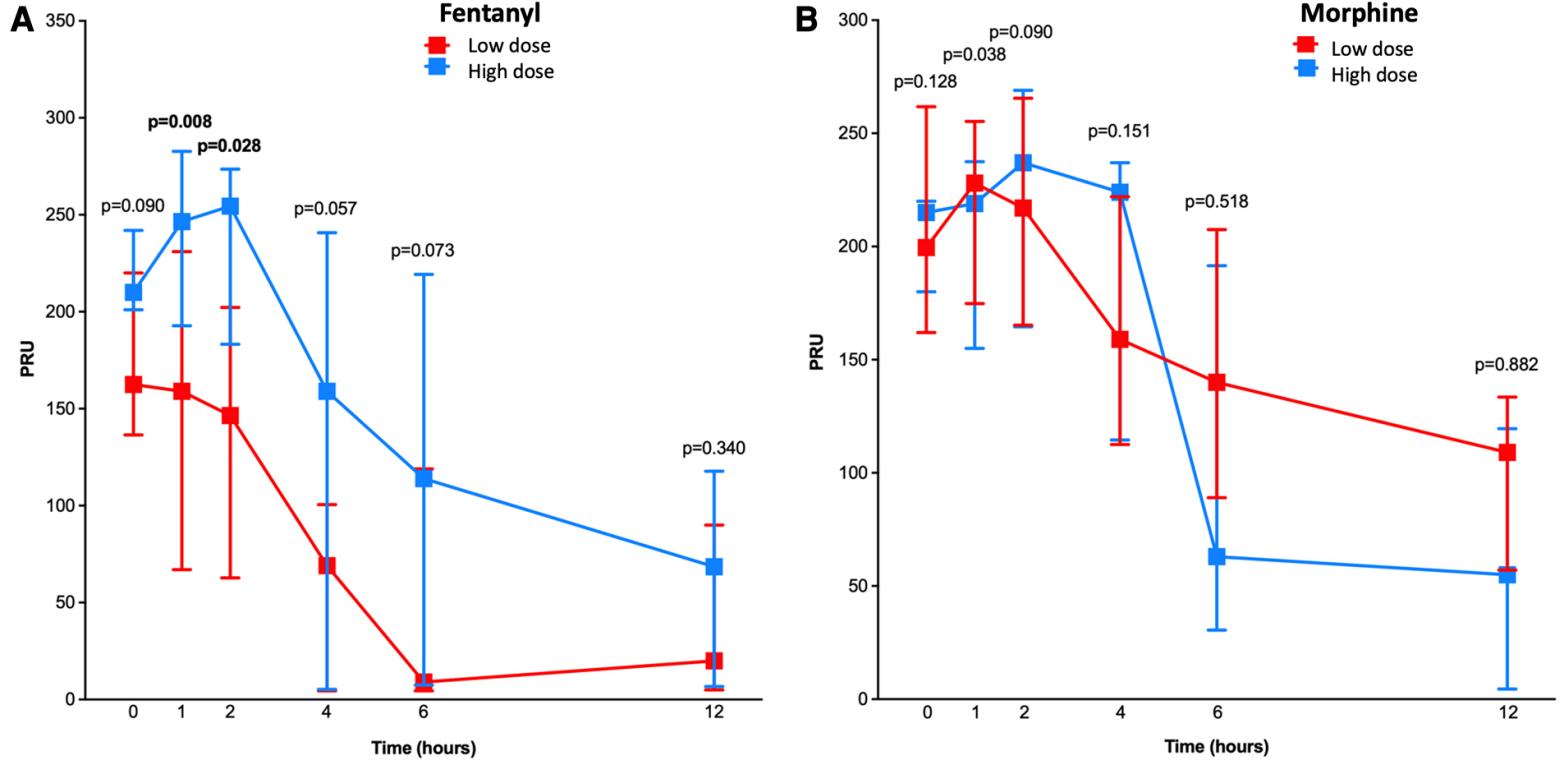
Pharmacodynamic assessment in patients treated with low vs. high doses of fentanyl and morphine. Line chart with P2Y_12_ reaction units (PRU) for low (in red) and high (in blue) doses of fentanyl (**A**) and morphine (**B**) at 1, 2, 4, 6, and 12 h after ticagrelor loading dose administration. *P*-values for differences between the two treatment groups are shown.

When stratified according to low vs. high doses of opioids received (Figure 1), PRU measured at 2 h was significantly lower among patients who received low, as compared to high, doses of fentanyl [147; interquartile range (IQR), 63–202; vs. 255; IQR, 183–274; *p* = 0.028], whereas no significant difference was found in those receiving low vs. high doses of morphine (217; IQR, 165–266 vs. 237; IQR, 165–269; *p* = 0.09). At 1 h, PRU values were significantly lower in patients treated with low vs. high doses of fentanyl (159; IQR, 67–231; vs. 247; IQR, 193–283; *p* = 0.008) (Figure 1). Overall, there was a signal suggesting lower PRU values in patients receiving low, as compared to high, doses of fentanyl at 4, 6 and 12 h, whereas no significant differences were observed in patients who received low vs. high doses of morphine (Figure 1).

When stratified by terciles of the total dose of opioids received (Supplementary Figure S1), PRU was significantly lower among patients in the lowest, as compared to those in the higher, fentanyl dose tercile group at 1 (159; IQR, 67–255; vs. 259; IQR, 213–287; *p* = 0.031), 2 (120; IQR, 52–216; vs. 255; IQR, 217–278; *p* = 0.041), and 6 (55; IQR, 5–119; vs. 128; IQR, 9–220; *p* = 0.039) hours after ticagrelor LD administration. No significant differences in PRU between lowest vs. highest dose tercile groups were found at any timepoint among patients who received morphine (Supplementary Figure S1).

### 3.3 Pharmacokinetic assessment

At 2 h, no significant associations were found between raw doses of fentanyl or morphine and plasma concentrations of ticagrelor and AR-C124910XX at 1 and 2 h following ticagrelor LD administration (Table 2). However, there were significant relationships between weight-weighted doses of fentanyl and morphine and plasma concentrations of ticagrelor (B, −1.39; 95% CI, −1.60–−1.18; *p* < 0.001 and B, −13.47; 95% CI, −17.26–−9.68; *p* < 0.001, respectively) and AR-C124910XX (B, −0.27; 95% CI, −0.31–−0.24; *p* < 0.001, and B, −1.10; 95% CI, −1.77–−0.43; *p* < 0.001, respectively) (Table 2). At 1 h, weight-weighted doses of fentanyl were significantly correlated with ticagrelor (B, 1.18; 95% CI, −1.34–−1.01; *p* < 0.001) and AR-C124910XX (B, −0.14; 95% CI, −0.16–−0.12; *p* < 0.001) plasma concentrations, whereas there was a significant correlation between weight-weighted doses of morphine and plasma concentrations of AR-C124910XX (B, 0.66; 95% CI, 0.28–1.04; *p* = 0.001), but not ticagrelor (B, 2.07; 95% CI, −0.99–5.13; *p* = 0.185) (Table 2).

**Figure 2:**
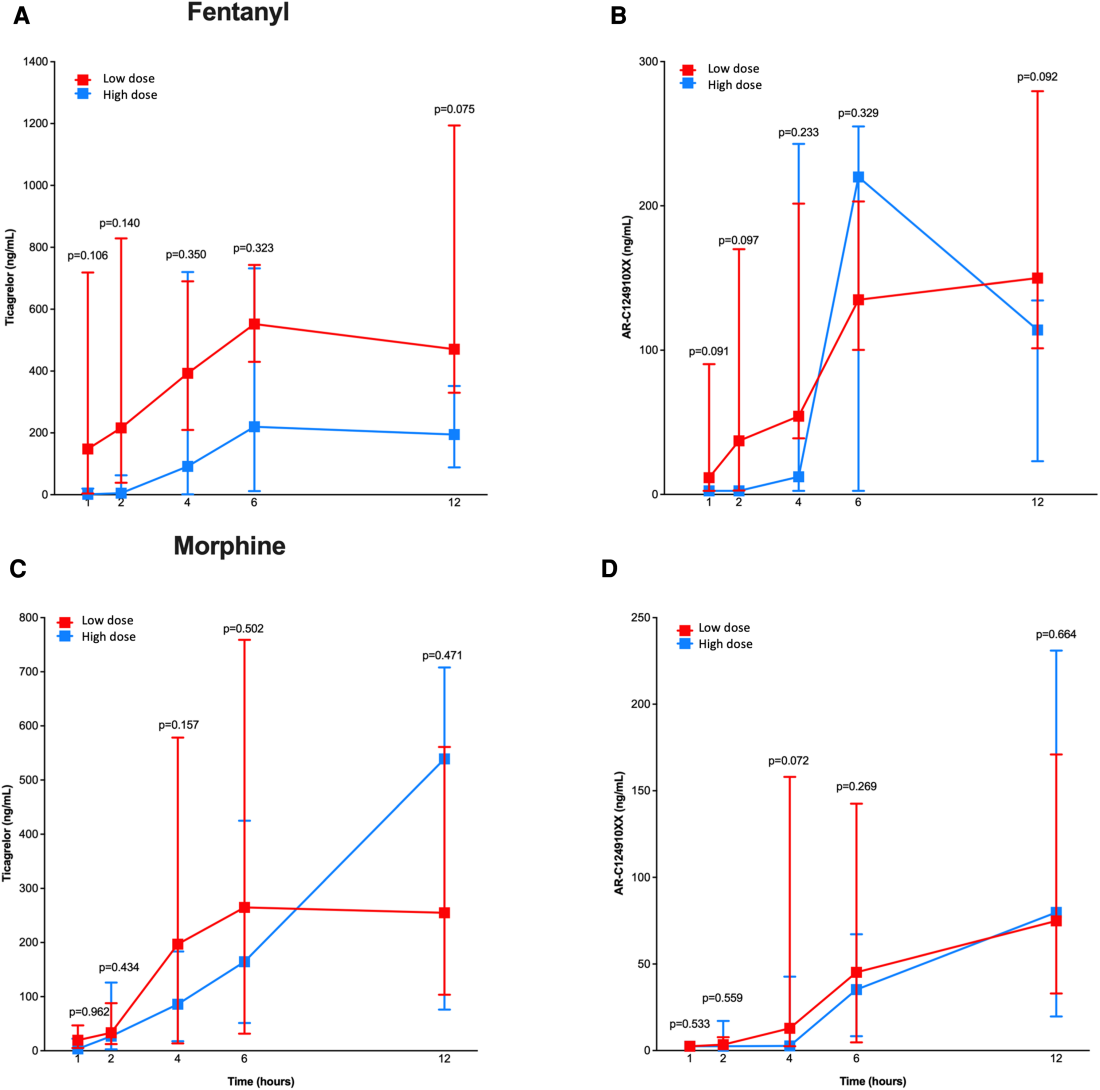
Pharmacokinetic assessment in patients treated with low vs. high doses of fentanyl and morphine. Line chart with pharmacokinetic profiles of ticagrelor (**A,C**) and AR-C124910XX (**B,D**) for low (in red) and high (in blue) doses of fentanyl (**A,B**) and morphine (**C,D**) at 1, 2, 4, 6 and 12 h after ticagrelor loading dose administration. *P*-values for differences between the two groups treatment are shown.

When stratified according to total intravenous opioid dose received (Figure 2), there were no significant differences between patients treated with low vs. high doses of fentanyl or morphine with regards to ticagrelor and AR-C124910XX plasma concentrations at 1, 2, 4, 6 and 12 h after ticagrelor pretreatment. However, we found a signal towards higher ticagrelor plasma concentrations among patients receiving lower, as compared to those who received higher, doses of fentanyl (Figure 2).

## 4 Discussion

In this *post hoc* subgroup analysis from the PERSEUS randomized trial, we found a significant correlation between both raw and weight-weighted doses of fentanyl, but not morphine, and ticagrelor-induced platelet inhibition among patients with STEMI treated with primary PCI and who received intravenous opioids for pain relief after P2Y_12_ inhibitor pretreatment with ticagrelor. To our knowledge, the present analysis is the first study demonstrating pharmacological differences between intravenous fentanyl and morphine with respect to platelet inhibition induced by a potent oral P2Y_12_ inhibitors according to the total dose of intravenous opioids received.

A robust body of randomized evidence suggests that platelet inhibition induced by potent orally administered P2Y_12_ receptor antagonists is affected by the concomitant use of opioids in the setting of STEMI (5, 6, 18, 19, 23). Intravenous opioids have been shown to delay gastro-intestinal drug absorption of oral P2Y_12_ inhibitors during the management of STEMI (24–26), which results in higher platelet reactivity and an increased risk for adverse ischemic and thrombotic events (4, 27, 28). Previous studies have suggested a potential direct association between total doses of intravenous opioids administered and platelet inhibition induced by potent oral P2Y_12_ inhibitors among symptomatic STEMI patients who underwent primary PCI (20, 21), and clinical research to further understand the underlying mechanisms for this drug-drug interaction between intravenous opioids and P2Y_12_ receptor inhibitors is still underway. A recent subgroup analysis from the MOVEMENT (“*Methylnaltrexone to Improve Platelet Inhibition of Ticagrelor in Morphine-Treated Patients With ST-Segment Elevation Myocardial Infarction”*) trial demonstrated a dose-dependent relationship between the total dose of morphine administered and platelet inhibition among STEMI patients, with higher morphine doses significantly reducing absorption and platelet inhibition induced by ticagrelor as compared to lower doses of morphine (20). In a subanalysis from the PACIFY (“*Platelet Aggregation with tiCagrelor Inhibition and FentanYl”*) trial (21), intravenous fentanyl was also shown to reduce ticagrelor absorption by a dose- and time-dependent attenuation of its platelet inhibitory effects among patients with chronic coronary syndromes undergoing PCI. However, no previous study has evaluated to date the differential impact of the total doses of fentanyl vs. morphine administered on pharmacokinetic and pharmacodynamic profiles of potent oral P2Y_12_ receptor antagonists among patients with STEMI undergoing primary PCI. This present subgroup analysis from the PERSEUS randomized trial demonstrates for the first time a dose-dependent association between total raw and weight-weighted doses of fentanyl, but not morphine, and the antiplatelet effects induced by ticagrelor pretreatment in the setting of primary PCI for STEMI, with greater platelet inhibition achieved with lower doses, as compared to higher doses, of fentanyl. These results are further supported by pharmacokinetic analyses demonstrating a dose-dependent delay in ticagrelor absorption, as suggested by lower plasma concentrations of ticagrelor and its active metabolite with higher doses of intravenous fentanyl administered. Our findings differ from those observed in a recent substudy from the MOVEMENT trial that found increased ticagrelor-induced platelet reactivity among patients with STEMI who received higher doses of morphine after ticagrelor pretreatment (20). These different results might be explained by significant differences between the two studies in total morphine doses administered, techniques used for pharmacodynamic assessment, and statistical methods.

The two aforementioned post-hoc studies showed a dose-dependent association between the amount of opioid received and altered ticagrelor pharmacology: one with morphine in ACS (20) and the other with fentanyl in stable coronary artery disease (21). However, it was not known whether the dose-dependent relationship of fentanyl on altered ticagrelor pharmacology also existed in ACS. As the PERSEUS trial was the first direct randomized comparison between intravenous fentanyl and morphine in STEMI patients, we reanalyzed this population to explore the association between fentanyl dose and ticagrelor-induced platelet inhibition in ACS. The present analysis provides novel insights into the existing drug-drug interaction between P2Y_12_ receptor inhibitors and intravenous opioids doses administered in the setting of STEMI. Pain relief during STEMI management is of paramount importance for patient comfort and to reduce sympathetic activation that causes vasoconstriction and increases cardiac workload (29, 30). Despite morphine has been consistently shown to reduce gastro-intestinal absorption, delay the onset of action, and decrease the antiplatelet effects of oral P2Y_12_ receptor antagonists in STEMI patients (5, 6, 18, 23), the use of intravenous opioids to relieve acute chest pain is still recommended (1, 31). Alternative strategies have been investigated to overcome the adverse effects of morphine on platelet inhibition induced by P2Y_12_ receptor antagonists but have not consistently shown improvements in pharmacokinetic and pharmacodynamic profiles of potent oral P2Y_12_ receptor inhibitors in STEMI patients (10, 32–35). Recently, in the ON-TIME 3 (“*Opioids aNd crushed Ticagrelor In Myocardial infarction Evaluation”*) randomized trial that compared treatment with fentanyl vs. intravenous paracetamol among STEMI patients undergoing primary PCI after ticagrelor pretreatment, there was no significant differences in ticagrelor-induced platelet inhibition at 2 h between treatment arms, despite higher plasma concentrations of ticagrelor at the start and immediately after primary PCI observed in patients who received paracetamol (16). The use of cangrelor, an intravenous P2Y_12_ antagonist with rapid onset and offset of action, results in consistent and potent P2Y_12_ receptor inhibition when administered in combination with ticagrelor and may represent an attractive alternative to bridge the gap until oral P2Y_12_ inhibitors achieve effective antiplatelet effects in STEMI patients undergoing primary PCI (12, 13, 36). However, to the best of our knowledge, no dedicated clinical trial has directly studied the impact of opioids on the pharmacodynamics and pharmacokinetics of cangrelor. Our study suggests that when intravenous opioids are needed to relieve acute pain in symptomatic STEMI patients who were pre-treated with ticagrelor, the use of lower, instead of higher, doses of fentanyl may accelerate ticagrelor absorption and achieve faster platelet inhibition compared to the use of morphine. These findings may be of relevant clinical interest given the lack of effective therapeutic alternatives to intravenous opioids for pain relief in the management of STEMI (16, 37). However, considering the post-hoc and non-prespecified design of the present study and the small number of patients included, these results are hypothesis-generating and a larger-scale dedicated randomized trial is needed to confirm our findings. Finally, the question of whether potential differences in dose-dependent pharmacological responses to ticagrelor pretreatment found in symptomatic patients with STEMI receiving intravenous fentanyl or morphine may translate into differential clinical outcomes remains to be determined.

The results of the present analysis should be interpreted in view of several limitations. First, this study is a *post hoc*, non-prespecified, subgroup analysis from the PERSEUS randomized trial, whose sample size was already small; its results should therefore be interpreted with caution and are hypothesis-generating concepts that warrant confirmation from larger-scale dedicated studies. Second, whereas patients were randomly allocated to fentanyl or morphine, we did not stratify randomization according to the dose of intravenous opioids administered. Third, we categorized patients who were randomized in the PERSEUS trial into different small subgroups according to low vs. high doses of opioids received. Considering the initial small sample size of the PERSEUS trial, this may have further reduced statistical power to compare individual ouctomes between treatment groups and prevented from analyzing other relevant outcomes investigated in the main analysis and in similar studies, such as the proportion of patients with HTPR, achievement of Thrombolysis In Myocardial Infarction grade 3 flow in the infarct-related artery prior to PCI, or ≥70% ST-segment elevation resolution after primary PCI (5, 15, 18, 21, 23). Finally, the results of this analysis may not be applicable to other oral P2Y_12_ receptor inhibitors than ticagrelor.

### 4.1 Conclusion

In patients with STEMI undergoing primary PCI after ticagrelor pretreatment and who have received intravenous opioids for acute pain relief, there was a dose-dependent relationship between the doses of intravenous fentanyl, but not morphine, administered and ticagrelor-induced platelet inhibition. These findings suggest that when intravenous opioids are needed to relieve acute pain in the management of STEMI, the use of lower, instead of higher, doses of fentanyl may accelerate ticagrelor absorption and achieve faster platelet inhibition compared to the use of morphine.

## Data Availability

The raw data supporting the conclusions of this article will be made available by the authors, without undue reservation.
