# Supplementary material for: Differential impact of fentanyl and morphine doses on ticagrelor-induced platelet inhibition in ST-segment elevation myocardial infarction: a subgroup analysis from the PERSEUS randomized trial
Source: Front Cardiovasc Med. 2024 Apr 2;11:1324641. doi: 10.3389/fcvm.2024.1324641 (PMC11018886; doi:10.3389/fcvm.2024.1324641)
Supplement: Supplementary file 1 [file Table1.docx]

Supplementary Material

**Supplementary material to: Differential impact of weighted-doses of fentanyl versus morphine on ticagrelor-induced platelet inhibition in ST-segment elevation myocardial infarction: a subgroup analysis from the PERSEUS randomized trial, by Garin D.**

# Supplementary Figures

## Supplementary Figure 1. Pharmacodynamic assessment in patients treated with fentanyl versus morphine and stratified by terciles of the total opioid dose received.


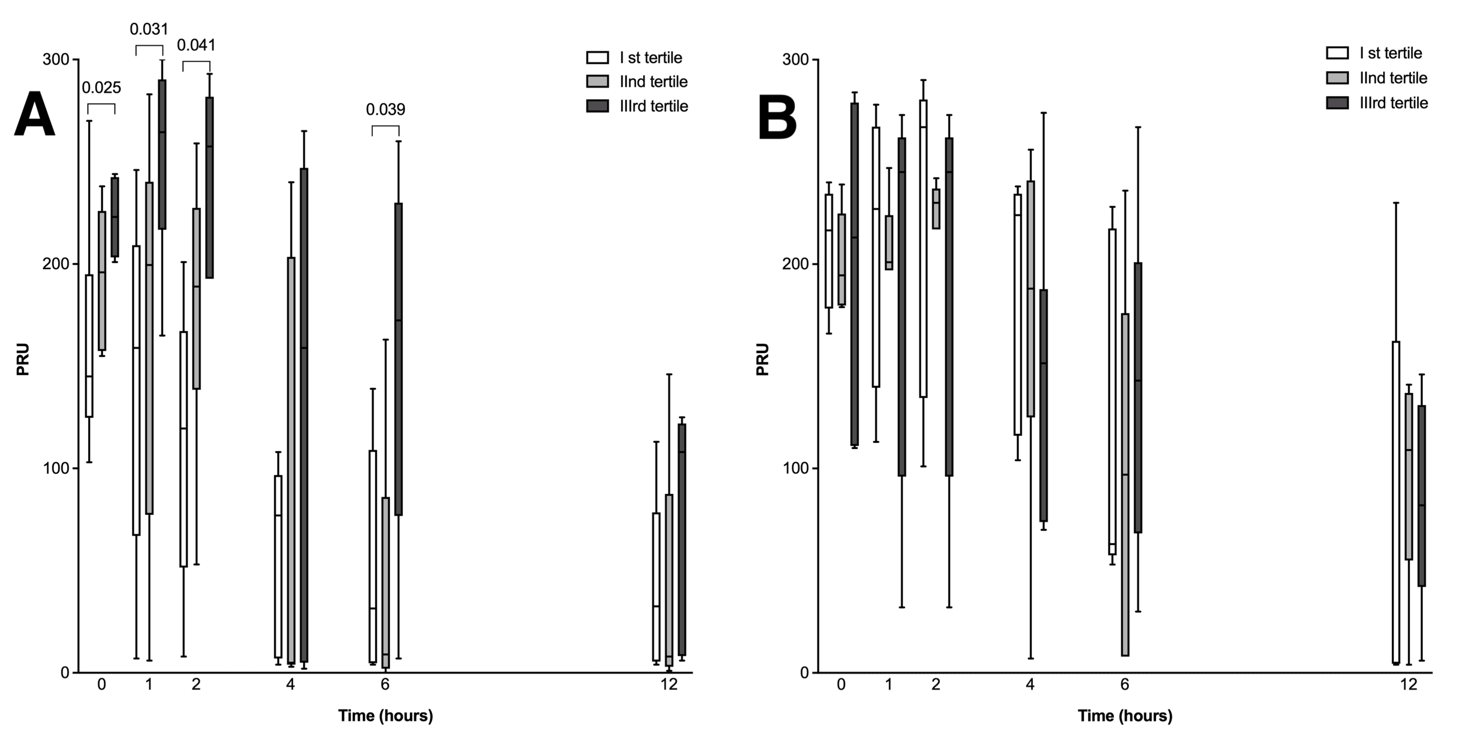


Box-plots with P2Y_12_ reaction units (PRU) at 1, 2, 4, 6 and 12 h after ticagrelor loading dose administration in patients treated with fentanyl (panel A) versus morphine (panel B) who were stratified by terciles according to the total opioid dose received. P-values <0.05 between tercile 1 and tercile 3 are shown. Remaining p values were non-significant.
